# A Machine Learning Approach for Monitoring and Classifying Healthcare Data—A Case of Emergency Department of KSA Hospitals

**DOI:** 10.3390/ijerph20064794

**Published:** 2023-03-08

**Authors:** Mahmoud Ragab, Faris Kateb, Mohammed W. Al-Rabia, Diaa Hamed, Turki Althaqafi, Abdullah S. AL-Malaise AL-Ghamdi

**Affiliations:** 1 Information Technology Department, Faculty of Computing and Information Technology, King Abdulaziz University, Jeddah 21589, Saudi Arabia; 2 Mathematics Department, Faculty of Science, Al-Azhar University, Naser City, Cairo 11884, Egypt; 3 Department of Medical Microbiology and Parasitology, Faculty of Medicine, King Abdulaziz University, Jeddah 21589, Saudi Arabia; 4 Health Promotion Center, King Abdulaziz University, Jeddah 21589, Saudi Arabia; 5 Mineral Resources and Rocks Department, Faculty of Earth Sciences, King Abdulaziz University, Jeddah 21589, Saudi Arabia; 6 Geology Department, Faculty of Science, Al-Azhar University, Naser City, Cairo 11884, Egypt; 7 Information Systems Department, HECI School, Dar Alhekma University, Jeddah 22246, Saudi Arabia; 8 Information Systems Department, Faculty of Computing and Information Technology, King Abdulaziz University, Jeddah 21589, Saudi Arabia

**Keywords:** machine learning, healthcare system, feature selection, metaheuristics, hyperparameter tuning, emergency department

## Abstract

The Emergency Departments (EDs), in hospitals located in a few important areas in Saudi Arabia, experience a heavy inflow of patients due to viral illnesses, pandemics, and even on a few special occasions events such as Hajj or Umrah, when pilgrims travel from one region to another with severe disease conditions. Apart from the EDs, it is critical to monitor the movements of patients from EDs to other wards inside the hospital or in the region. This is to track the spread of viral illnesses that require more attention. In this scenario, Machine Learning (ML) algorithms can be used to classify the data into many classes and track the target audience. The current research article presents a Machine Learning-based Medical Data Monitoring and Classification Model for the EDs of the KSA hospitals and is named MLMDMC-ED technique. The most important aim of the proposed MLMDMC-ED technique is to monitor and track the patient’s visits to the EDs, the treatment given to them based on the Canadian Emergency Department Triage and Acuity Scale (CTAS), and their Length Of Stay (LOS) in the hospital, based on their treatment requirements. A patient’s clinical history is crucial in terms of making decisions during health emergencies or pandemics. So, the data should be processed so that it can be classified and visualized in different formats using the ML technique. The current research work aims at extracting the textual features from the patients’ data using the metaheuristic Non-Defeatable Genetic Algorithm II (NSGA II). The data, collected from the hospitals, are classified using the Graph Convolutional Network (GCN) model. Grey Wolf Optimizer (GWO) is exploited for fine-tuning the parameters to optimize the performance of the GCN model. The proposed MLMDMC-ED technique was experimentally validated on the healthcare data and the outcomes indicated the improvements of the MLMDMC-ED technique over other models with a maximum accuracy of 91.87%.

## 1. Introduction

**Figure 1:**
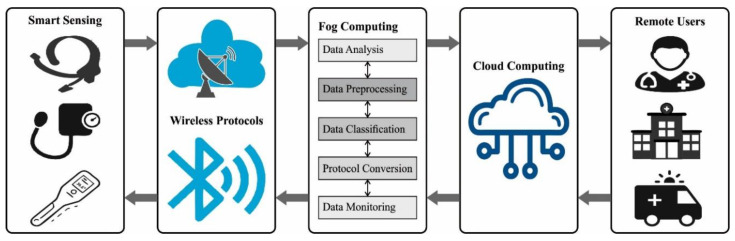
Emergency Department Process.

The demand for medical care has dramatically increased, especially after the COVID-19 pandemic. So, the problem of managing the Emergency Departments (ED) in hospitals, especially the inflow of patients, is certainly a major problem that needs to be carefully alleviated [1]. If it is not appropriately managed, then this might result in overcrowding, and a degradation in the quality of services. EDs are the first access point for patients who are transported with urgent concerns such as injury and sudden illness without any history of ailments [2]. This makes the functioning of EDs increasingly difficult due to resource constraints for the treatment level required, unpredicted time of patients’ arrival, and various requirements of the patients [3]. Figure 1 shows the block diagram of the emergency department process in which the smart sensing devices are enabled to collect the patient’s data. Then, the data gets transferred to both fog and cloud computing users via wireless connections for data analysis. Finally, the examined data are passed on to remote users such as physicians, hospital authorities, etc.

The demand for ED services has increased gradually over the past few years, especially after COVID-19. Consequently, the EDs are confronted with multiple issues and heavy pressure, produced by a high inflow of patients, which places them among the most-crowded departments of the hospital. Various research conducted earlier on ED found that the department encounters a large number of difficulties in satisfying the mission of the organization [4]. Therefore, it is crucial to develop a precise forecasting model for EDs for effective crowd management and proper optimization of the available resources [5]. The increased utilization of Electronic Health Records (EHR) systems has brought extraordinary opportunities for the medical informatics field [6]. Various research investigations have been conducted using this information on different tasks such as comparative effectiveness research, predictive modelling, and disease subtyping. The Machine Learning (ML) approach is a traditional tool for executing this task [7].

ML is a sub-field of Artificial Intelligence, and this technique represents the capability of IT systems to individually find solutions for problems via pattern recognition in the databases [8]. The ML techniques allow the IT systems to identify the patterns based on the existing technique and the dataset to design a novel satisfactory solution. The metaheuristic techniques aim to identify the optimal solution, from all the probable solutions to the optimization issue under consideration [9]. They determine the effective solutions and carry out a sequence of functions to find a specific effective solution. The metaheuristics techniques operate on a representation or encoding of a solution. This object can be stored in computer memory and conveniently manipulated by different operators, employed by the metaheuristic technique [10]. Metaheuristics have been demonstrated by the scientific community as a viable, and often superior alternative to the traditional (exact) methods of mixed-integer optimization such as the branch and bound, and dynamic programming.

Various studies have used ML models for the examination of healthcare data. In the literature [11], an emergency patient classification method was proposed based on an SVM classifier. The proposed method can be executed as a web application, expressed in PHP, and runs on the MySQL dataset. In this study, the GIS technique was utilized for analyzing the spatial data and generating relevant reports. The presented method categorized the emergency patients as distinct groups based on their severity and standard government. In the study conducted earlier [12], a Deep Belief Network (DBN) was proposed to overcome the issues faced in patient attendance disposals from the Accident and Emergency (A&E) department. The PSO technique was utilized in this study to fine-tune the hyperparameters utilized for the DBN model. The PSO technique helped in achieving an easy, simple, and comparatively fast convergence rate with a satisfactory solution. To be specific, the newly established Randomly Occurring Distributed Delayed PSO (RODDPSO) technique was able to attain the optimum solutions. The enhanced early convergence characteristic was used for the optimization of the hyperparameters involved in DBN.

Krämer et al. [13] developed a method for the classification of in-patient admissions, based on initial patient analysis, as either emergency care and selective care or predictive urgency, in the form of numerical values. The authors utilized the supervised ML systems and trained the method using physician-expert judgment. In this method, an urgency value is applied to all the relevant analyses, quoted in the ICD catalogue. The value was simply applied to the predefined hospital datasets. The findings offered a fundamental framework for strategy makers to create incentives for the clinics to minimize the count of incorrect ED admissions. Harrou et al. [14] presented an effectual system for forecasting continuous visits to the ED, utilizing Variational AutoEncoder (VAE) technique. Certainly, the VAE technique, as a DL-based method, obtained special attention in the modelling and extraction of the features. This is attributed to their distribution-free assumption and maximum non-linear calculation. 

Kamruzzaman et al. [15] proposed a Fuzzy-Assisted ML framework (F-AMLF) to identify the method for the mitigation of costs incurred upon device resources, while at the same time, retaining the efficacy limitation. In this method, the patients might submit their demands for healthcare in a fuzzy-assisted fog computing system. The proposed technique made use of fuzzy logic to evaluate the computing ability required to preserve the fog and cloud projections. Raza et al. [16] designed a novel end-to-end architecture in a federated setting for ECG-based healthcare with the help of deep Convolutional Neural Networks (CNN) and explainable Artificial Intelligence (XAI). Additionally, the presented architecture successfully categorized diverse arrhythmias using the classifier and Autoencoder, based on CNN.

Chen et al. [17] introduced a DL-based ECG signal super-resolution framework (named SRECG) to improve the low-resolution ECG signals through equal consideration of the performance. In this study, a DL-based high-resolution multiclass classifier (HMC) of the CAs was used. Newaz et al. [18] proposed HealthGuard, a novel ML-based security framework to identify malicious activities in SHS. This framework i.e., HealthGuard observes the major signs of the connected device of SHS and relates the vitals of the human body to understand any changes in them and differentiate the benign activities from the malignant ones. HealthGuard exploited four different ML-based detection methods (such as Decision Tree, Artificial Neural Network, k-Nearest Neighbor, and Random Forest) to identify the malicious activities in SHS. In a real-time scenario, the author [19] re-defined the SE system of securely outsourcing the electrocardiogram (ECG) data in untrusted BSN environments. If the ECG data are outsourced for disease classification based on the ML algorithm, it can be inferred that the classical SE scheme may not be the correct design. In the literature [20,21,22,23,24], the authors introduced a Learning-based Deep-Q-Network to reduce malware attacks, when dealing with healthcare data. This technique examined the medical data in multiple layers, based on a Q-learning model that assists in minimizing the intermediate attacks with less complexity. 

Though several models are available in the literature, only a few works have focused on the feature selection process. At the same time, owing to continuous refinement of the existing models, there is a rapid increase in the number of parameters in DL models too, which in turn results in model overfitting. At the same time, different hyperparameters exert a significant impact on the efficiency of the CNN model. Particularly, hyperparameters such as epoch count, batch size, and the learning rate selection are essential to attain effectual outcomes. Since the trial-and-error method for hyperparameter tuning is a tedious and erroneous process, metaheuristic algorithms are applied.

The current research article presents a Machine Learning-based Medical Data Monitoring and Classification Model for the EDs in the hospitals of KSA and the model is abbreviated as the MLMDMC-ED technique. The proposed model aims to extract the textual features from the patients’ data using the metaheuristic Non-Defeatable Genetic Algorithm II (NSGA II). The data, collected from the hospitals, is then classified using the Graph Convolutional Network (GCN) method. Further, Grey Wolf Optimizer (GWO) method is exploited as a parameter-tuning process in this study to optimize the performance of the GCN model. The proposed MLMDMC-ED technique was experimentally validated using the healthcare data under different measures. The experimental outcomes confirmed the improvements of the proposed MLMDMC-ED technique over other models. 

## 2. The Proposed Model

**Figure 2:**
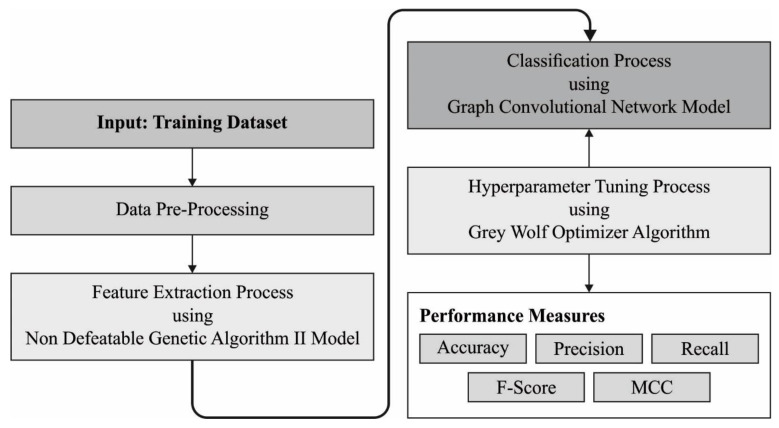
Working procedure of the MLMDMC-ED system.

The current research article presents the Machine Learning-based Medical Data Monitoring and Classification model for the EDs of the KSA hospitals, abbreviated as the MLMDMC-ED technique. The most important aim of the proposed technique is to monitor and track the patient’s visits to the EDs and the treatment given to them at the hospital based on CTAS and LOS as per the patients’ conditions. Patients’ data are crucial in making important clinical decisions during health emergencies or pandemics. Initially, the data are pre-processed through a min-max normalization approach, which scales the input data in the range of [0, 1]. It is important for the data to be appropriate to easily classify and use under many visualization formats using the ML model. The presented model exploited the NSGA II technique to choose the features. For classification, the GWO with GCN model is employed in this work. Figure 2 exhibits the working procedure of the proposed MLMDMC-ED system.

### 2.1. Feature Selection Using NSGA II Approach

Non-Defeatable Genetic Algorithm II (NSGA II) is one of the well-known and effective multi-objective optimization techniques. This method is the most powerful and fastest optimization technique with less operating difficulty than the rest of the techniques [25]. This approach concludes that a model has an optimum range with respect to changing the objective function and it also provides freedom to select the desired design amongst the available optimum designs. In the presented method, the conservation of dispersion and elitism is regarded concurrently. In all the steps of this technique, a novel population is selected based on the principles of dominance, utilization of the population ranking, and elitism in the overall solution steps. Amongst the available answers, the best and the undefeated answer is carefully chosen, thereby returning to the upcoming step.

**Figure 3:**
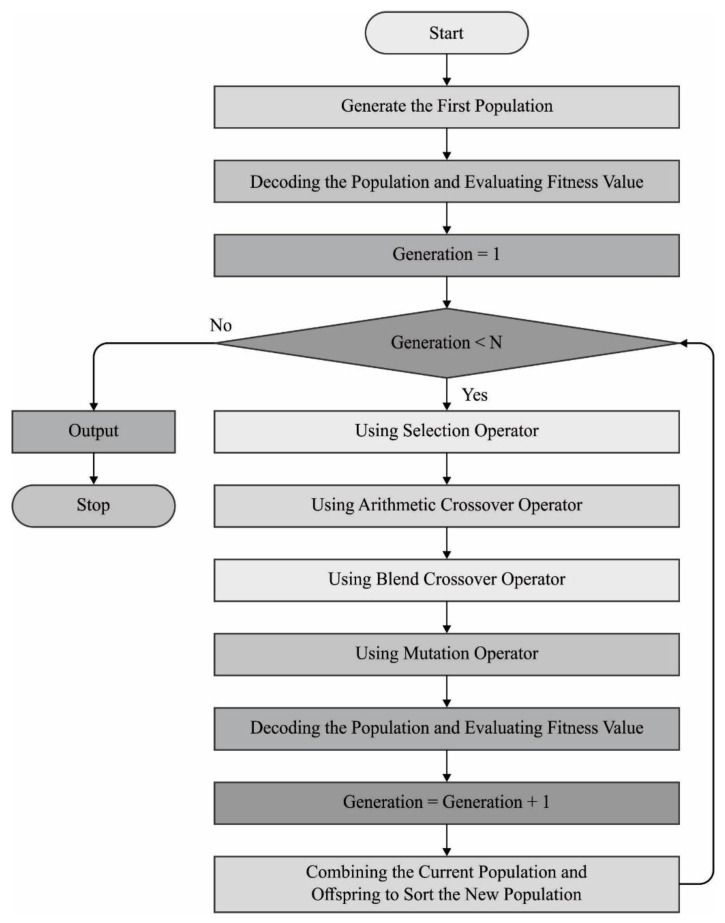
Flowchart of NSGA II Algorithm.

When two objective functions such as $f_{1}$ and $f_{2}$ exist, the answer x beats the answer y, thus $f_{1}\left(x\right)\geq f_{1}\left(y\right)$ and $f_{2}\left(x\right)\geq f_{2}\left(y\right)$ or $f_{1}\left(x\right)>f_{1}\left(y\right)$ and $f_{2}\left(x\right)>f_{2}\left(y\right)$. Similarly, an idea named ‘congestion distance’ is utilized to monitor the appropriate density distribution of each answer. In general, all the answers are compared to the rest of the answers in the population to determine whether an answer is defeated and accordingly arrange a population of size n, depending on the non-defeat level. At last, there exist many solutions and any of them may overcome the other one; thus, this solution forms an initial boundary with invincible boundaries. This answer is then passed on to set $F_{1}$. To define the answer in the following boundary, the answer in the initial boundary is disregarded as a temporary one. The abovementioned procedure is reiterated; this time, the answer is transported to $the\;F_{2}$ set and takes the second position. Figure 3 show Flowchart of NSGA II Algorithm.

This procedure is repeated for each unanswered question in the population. Indeed, arranging non-defeat is a process to accomplish the best answer. Further, the model of diversity seeks to preserve the breadth and diversity of the answer. In the presented method, this can be performed by swarming distance in such a way. This raises the dispersion and diversity of the attained answer.

(1) $$CD\left(X^{1}\right)=CD\left(X^{S}\right)=\infty ,$$

(2) $$CD\left(X^{i}\right)=\left[\frac{Z_{1}\left(X^{i+1}\right)-Z_{1}\left(X^{i-1}\right)}{Z_{1}\left(X^{S}\right)-Z_{1}\left(X^{1}\right)}\right]+\left[\frac{Z_{2}\left(X^{i+1}\right)-Z_{2}\left(X^{i-1}\right)}{Z_{2}\left(X^{S}\right)-Z_{2}\left(X^{1}\right)}\right],\;i=2,\;\ldots ,S-1.$$

In the abovementioned relationship, $CD\left(X\right)$ denotes the amount of congestion distance for $X^{i}$’s answer. Afterwards, both integration and the un-defeated sort are done as shown in steps, 7 and 8. 

According to step 10, the swarm distance condition is utilized for the creation of a subset of the final undefeated set, because of the succeeding increase in the population size:

Step 1. Generate a primary population $P_{0}$ of size N with a random answer and set t=0,
Step 2. When the stopping criteria are not satisfied, return to $P_{t},$
Step 3. By applying the binary selection operators, choose N parents from the population $P_{t},$
Step 4. Using the mutation and intersection operators on $the\;P_{t}$ population, produce a population of $Q_{t}$ children to size N
Step 5. Set $R_{t}=P_{t}UQ_{t},$
Step 6. Utilize the invincible ranking model to determine the Pareto $F_{i}$ set in the $R_{t}$ population.
Step 7. Set $P_{t+1}=\emptyset ,i=1,$
Step 8. Until $\left|P_{t+1}\left|+\right|F_{i}\right|<N$:
  (a) Add the answer of the set $F_{i}$ to the population $P_{t+1},$ and
  (b) Put i=i+1.
Step 9. Sort the $F_{i}$ set to answer in the descending sequence of congestion
Step 10. Size $N-\left|p\right|$ Transmit from initial answer $F_{i}$ to $P_{t+1}$ population, and
Step 11. Set t=t+1 and return to Step 2.

In the proposed approach, the fitness function is used to balance the classification accuracy (maximum) whereas the number of selected features, in every solution (minimum) attained through this selected feature, is determined as follows.

(3) $$Fitness=\alpha \gamma_{R}\left(D\right)+\beta \frac{\left|R\right|}{\left|C\right|}$$

Here, $\gamma_{R}\left(D\right)$ denotes the classification error rate. $\left|R\right|$ indicates the cardinality of the selected subset and $\left|C\right|$ represents the overall number of features in the data and the variables α and β correspond to the significance of the classification quality and the subset length respectively; ∈ [1, 0] and β=1−α.

### 2.2. Medical Data Classification Using the GCN Model

For the medical data classification process, the GCN model is utilized in this study. GCN is a multilayer neural network that acts on the graphs directly and produces the embedding vectors based on the neighborhood’s property [26]. Then, a two-stage GCN is utilized for the study. In the literature, Yao et al. utilized it to execute the text classification tasks:

(4) $$\hat{A}=D^{-1/2}AD^{-1/2}$$

Here, D denotes the degree of matrix, A. Hence, the output of the initial layer is the novel feature matrix $E_{1}$ or the word embedding, which is evaluated as follows

(5) $$E_{1}=ReLU\left(\hat{A}XW_{0}\right)$$

Here, X denotes the input feature matrix, $W_{0}$ indicates the initial weight, and *ReLU* is utilized as an activation function. The next layer is fed to softmax classification. The number of nodes is similar to the number of labels in the following layer. Hence, the output (*O*) is evaluated using the following expression:

(6) $$E_{2}=\hat{A}E_{1}W_{1}$$

(7) $$O=softmax\left(E_{2}\right)$$

$E_{2}$ indicates the embedding and novel feature matrix for the next layer, and $W_{1}$ denotes the weight of the initial layer.

### 2.3. Hyperparameter Tuning Using GWO Algorithm

The GWO algorithm is exploited in this study as a parameter-tuning process to optimize the performance of the GCN model. Mirjalili et al. [27] developed the GWO technique by simulating the leadership position and hunting strategies of the Grey-Wolves. The GWO technique functions based on flexibility, simplicity, and the avoidance of local optimization, while it also handles multi-variate and non-linear functions. The presented approach depends on the equation; it alters the random response produced during all the iterations which enhance the objective function. Finally, Alpha Wolf, a better primary solution, is obtained, whereby the outcome is the maximum expected income with a minimum investment risk. Grey-Wolves predominantly prefer to live in groups. 

In a group, the leader consists of a male and a female named α. The Alpha is mainly accountable for the decisions regarding hunting, when to wake up, where to sleep, etc. The decision of the Alpha is to interact with the groups, while some democratic behaviors are observed in which the α follows another wolf in the group. In the community, the entire herd endorses the α. The Alpha wolf can be called the dominant wolf since its command should be implemented by the group. The Alpha wolf is allowed to mate with the herd’s females. Beta is the wolf that assists the α in making the decisions or other decisions for the herd. β performs the α’s command through a herd and provides feedback to α. The Omega (ω) wolf is the least minimum class in the GW hierarchy. The Delta wolves are subjected to α and β. The optimized solution can be attained by distinct kinds of the wolf $\left(\alpha ,\;\beta \;and\;\delta \right)$ agents in the GWO. The new position of a single wolf, in encircling its prey, is defined by the following equations.

(8) $$G\left(t+1\right)=G_{p}\left(t\right)-A\cdot D,D=\left|C\cdot G_{p}\left(t\right)-G\left(t\right)\right|$$

(9) $$A=a\times \left(2\times r_{1}-1\right),\;C=2\times r_{2}$$

Here, t specifies the present iteration and A and C denote the coefficient vectors. A represents a coefficient that lies in the range of 2 to 0 and sum is a random number that differs from 0 to 1. 

The position of the wolf is upgraded as follows:

(10) $$G\left(t+1\right)=\frac{G_{1}+G_{2}+G_{3}}{3}$$

G1, G2, and G3 correspond to dissimilar types of grey wolves (α, β, and δ) and are evaluated using the following expressions [28]:

(11) $$D_{a}=\left|C_{1}\cdot G_{\alpha }-G\right|$$

(12) $$D_{\beta }=\left|C_{1}\cdot G_{\beta }-G\right|$$

(13) $$D_{\delta }=\left|C_{1}\cdot G_{\delta }-G\right|$$

(14) $$G_{1}=G_{\alpha }-A_{1}\cdot D_{\alpha }$$

(15) $$G_{2}=G_{\beta }-A_{2}\cdot D_{\beta }$$

(16) $$G_{3}=G_{\delta }-A_{3}\cdot D_{\delta }$$

The Gray Wolf group finishes the hunt by attacking and encircling their prey. To obtain this, they should go to a certain location to approach the prey while the attack can be accomplished by changing the values from 2 to 0. To accomplish an improved classification performance, the GWO algorithm derives a fitness function. It uses a positive integer to denote the good performance of the candidate solution. In the presented model, the reduction in classification error rate is viewed as the fitness function.

(17) $$fitness\left(x_{i}\right)=ClassifierErrorRate\left(x_{i}\right)=\frac{number\;of\;misclassified\;samples}{Total\;number\;of\;samples}*100$$

## 3. Results and Discussion

In this section, the performance of the proposed MLMDMC-ED technique was validated using two datasets such as the Cleveland dataset (https://archive.ics.uci.edu/ml/datasets/heart+disease (accessed on 6 February 2023)) and the Statlog dataset (https://archive.ics.uci.edu/ml/datasets/statlog+(heart) (accessed on 6 February 2023)). The proposed model was simulated using the Python tool. Table 1 illustrates the details of both datasets used in the study.

**Figure 4:**
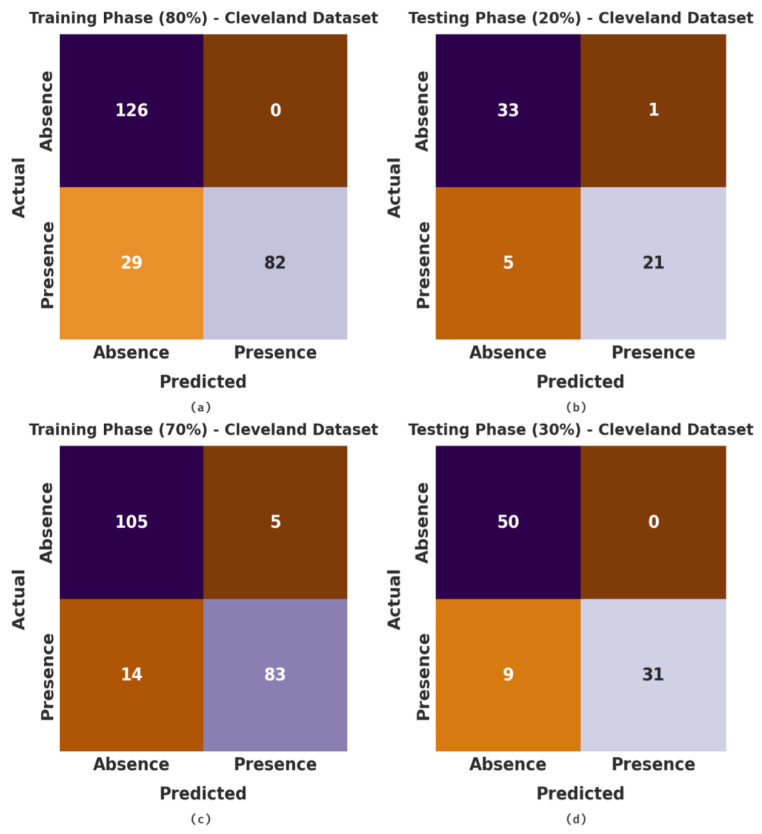
Confusion matrices of MLMDMC-ED method on Cleveland dataset (**a**,**b**) TR and TS database of 80:20 and (**c**,**d**) TR and TS database of 70:30.

The proposed model considered the following attributes: Age, sex, cp, trestbps, chol, fbs, restecg, exang, Thalach, oldpeak, slope, ca, and thal. The confusion matrices, generated by the proposed MLMDMC-ED technique on the Cleveland dataset, are portrayed in Figure 4. With 80% of the TR database, the proposed MLMDMC-ED model classified 126 samples under the absence class and 82 samples under the presence class. In addition, with 20% of the TS database, the presented model positioned 33 samples under the absence class and 21 samples under the presence class. In addition, with 70% of the TR database, the proposed MLMDMC-ED approach recognized 105 samples as absence class and 83 samples as presence class.

**Figure 5:**
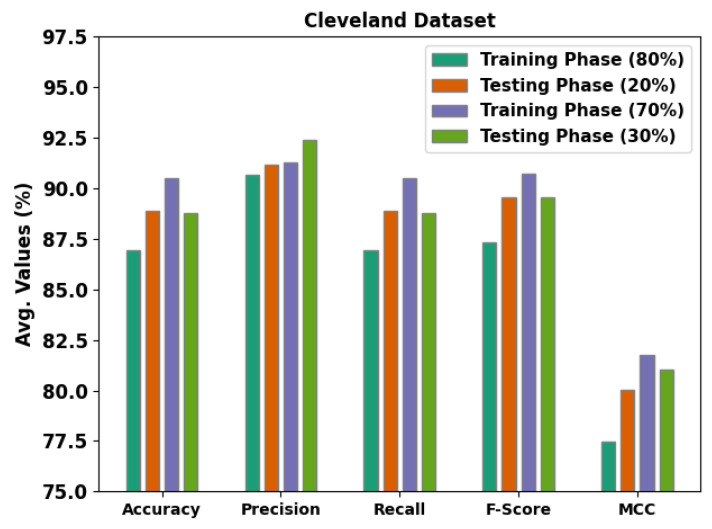
Average analysis results of the MLMDMC-ED algorithm on the Cleveland dataset.

Table 2 and Figure 5 portray the detection results obtained by the proposed MLMDMC-ED method on the Cleveland dataset. On 80% of the TR database, the proposed model gained the average $accu_{bal}$, $prec_{n}$, $reca_{l}$, $F_{score}$, and MCC values of 86.94%, 90.65%, 86.94%, 87.33%, and 77.49%, respectively. Moreover, on 20% of the TS database, the presented model gained the average $accu_{bal}$, $prec_{n}$, $reca_{l}$, $F_{score}$, and MCC values of 88.91%, 91.15%, 88.91%, 89.58%, and 80.03% respectively. Furthermore, on 70% of the TR database, the MLMDMC-ED model attained the average $accu_{bal}$, $prec_{n}$, $reca_{l}$, $F_{score}$, and MCC values of 90.51%, 91.28%, 90.51%, 90.72%, and 81.78% correspondingly.

**Figure 6:**
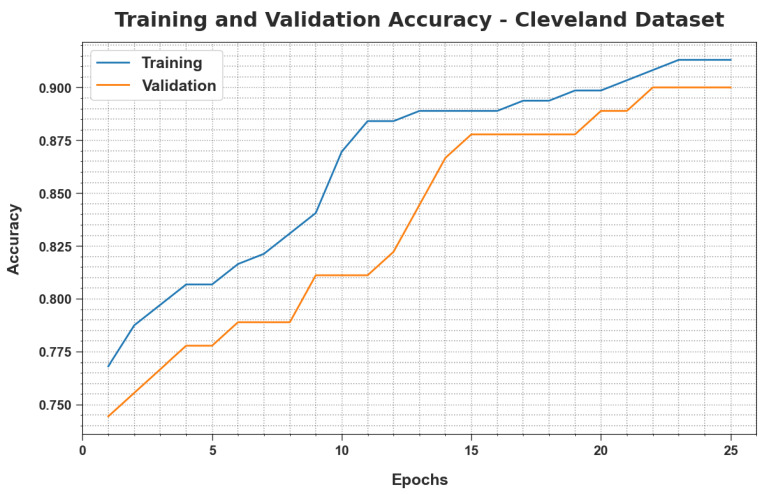
$TR_{acc}$ and $VL_{acc}$ analyse the results of the MLMDMC-ED algorithm on the Cleveland dataset.

The Training Accuracy ($TR_{acc}$) and Validation Accuracy ($VL_{acc}$) values, obtained by the proposed MLMDMC-EDR method upon the Cleveland database, are illustrated in Figure 6. The outcomes exhibit that the MLMDMC-EDR approach reached the highest $TR_{acc}$ and $VL_{acc}$ values whereas the $VL_{acc}$ values were higher than the $TR_{acc}$ values.

**Figure 7:**
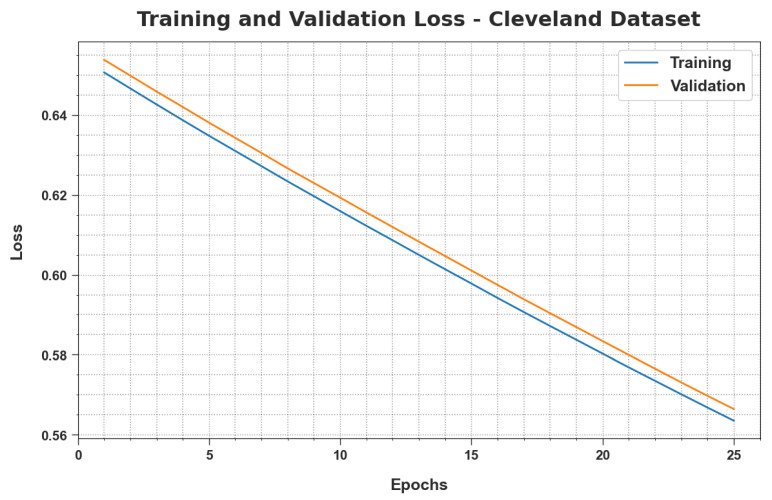
$TR_{loss}$ and $VL_{loss}$ analysis results of the MLMDMC-ED algorithm on the Cleveland dataset.

The Training Loss ($TR_{loss}$) and Validation Loss ($VL_{loss}$) values, attained by the proposed MLMDMC-EDR approach on the Cleveland database, are illustrated in Figure 7. The experimental outcomes infer that the MLMDMC-EDR algorithm accomplished the minimal $TR_{loss}$ and $VL_{loss}$ values while the $VL_{loss}$ values were less than the $TR_{loss}$ values.

**Figure 8:**
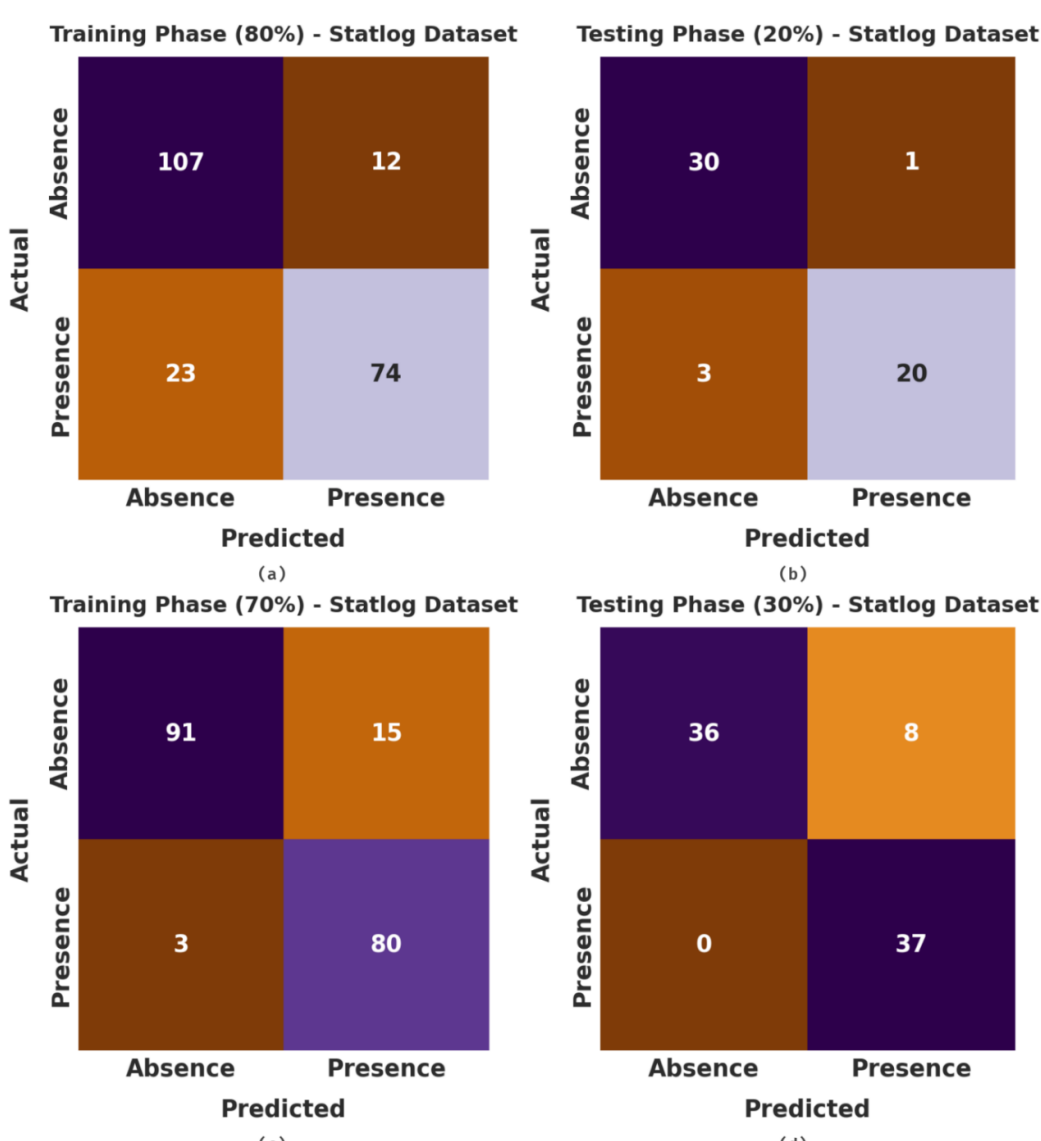
Confusion matrices of MLMDMC-ED method under Statlog dataset (**a**,**b**) TR and TS database of 80:20 and (**c**,**d**) TR and TS database of 70:30.

The confusion matrices, generated by the proposed MLMDMC-ED model on the Statlog dataset, are portrayed in Figure 8. With 80% of the TR database, the MLMDMC-ED model classified 107 samples under the absence class and 74 samples under the presence class. Furthermore, with 20% of the TS database, the proposed MLMDMC-ED model segregated 30 samples under the absence class and 20 samples under the presence class. In addition, with 70% of the TR database, the MLMDMC-ED model identified 91 samples as absence class and 80 samples as presence class.

**Figure 9:**
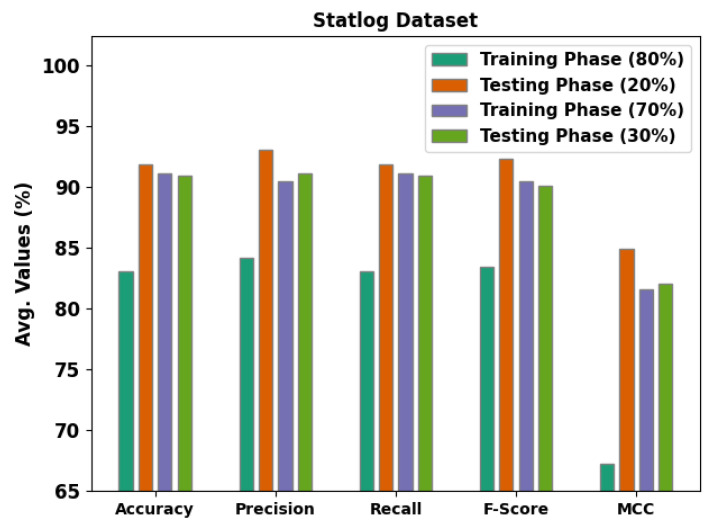
Average analysis results of the MLMDMC-ED algorithm upon Statlog dataset.

Table 3 and Figure 9 show the detection outcomes obtained by the proposed MLMDMC-ED approach on the Statlog dataset. On 80% of the TR database, the MLMDMC-ED model obtained the average $accu_{bal}$, $prec_{n}$, $reca_{l}$, $F_{score}$, and MCC values of 83.10%, 984.18%, 83.10%, 83.41%, and 67.27%, correspondingly. Furthermore, on 20% of the TS database, the proposed MLMDMC-ED model gained the average $accu_{bal}$, $prec_{n}$, $reca_{l}$, $F_{score}$, and MCC values of 91.87%, 93.07%, 91.87%, 92.33%, and 84.93%, correspondingly. Moreover, on 70% of TR databases, the presented MLMDMC-ED approach attained the average $accu_{bal}$, $prec_{n}$, $reca_{l}$, $F_{score}$, and MCC values of 91.12%, 90.51%, 91.12%, 90.44%, and 81.62%, correspondingly.

**Figure 10:**
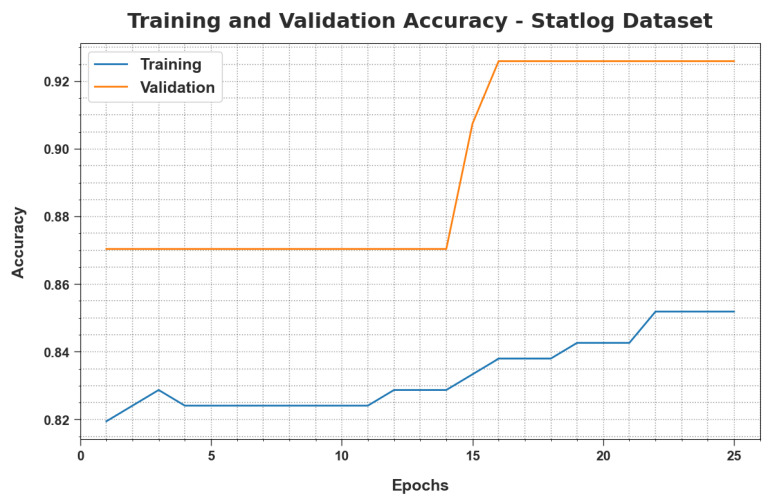
$TR_{acc}$ and $VL_{acc}$ analyse the results of the MLMDMC-ED algorithm on the Statlog dataset.

$TR_{acc}$ and $VL_{acc}$ values, obtained by the MLMDMC-EDR method on the Statlog database, are illustrated in Figure 10. The experimental outcomes confirm that the MLMDMC-EDR approach achieved the maximum $TR_{acc}$ and $VL_{acc}$ values while the $VL_{acc}$ values were higher than the $TR_{acc}$ values.

**Figure 11:**
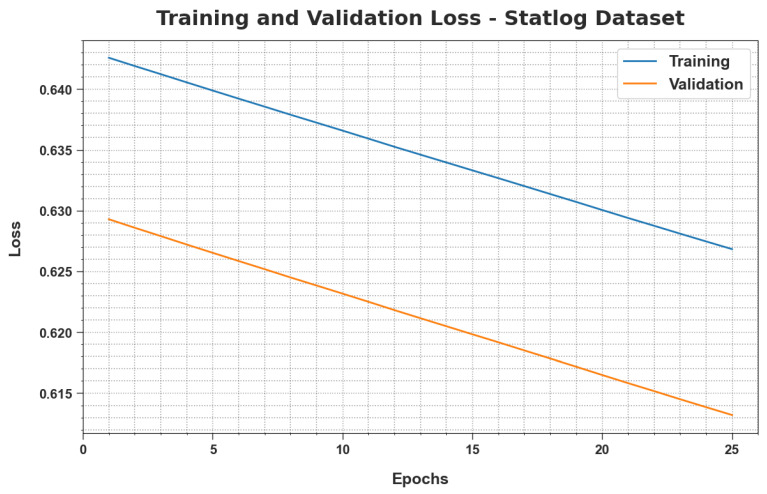
$TR_{loss}$ and $VL_{loss}$ analyses results of the MLMDMC-ED algorithm on the Statlog dataset.

$TR_{loss}$ and $VL_{loss}$ values, attained by the proposed MLMDMC-EDR approach upon Statlog database, are demonstrated in Figure 11. The experimental outcomes demonstrate that the proposed MLMDMC-EDR algorithm attained the minimum $TR_{loss}$ and $VL_{loss}$ values whereas the $VL_{loss}$ values were less than the $TR_{loss}$ values.

**Figure 12:**
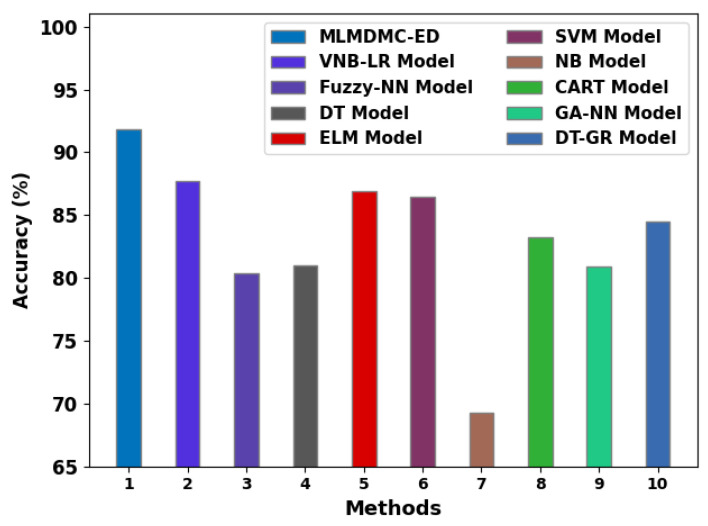
Accuracy analysis outcomes of the proposed MLMDMC-ED algorithm and other recent algorithms.

In this final stage, a widespread comparative analysis was conducted between the proposed MLMDMC-ED algorithm and other existing approaches and the results are shown in Table 4 and Figure 12 [29]. The outcomes indicate the enhanced performance of the MLMDMC-ED model with a maximum $accu_{y}$ of 91.87%. On the contrary, the VNB-LR, fuzzy-NN, DT, ELM, SVM, NB, CART, GA-NN, and DT-GR models achieved the least $accu_{y}$ values. Thus, the MLMDMC-ED model can be employed for accurate ransomware detection. The enhanced performance of the proposed model is attributed to the design of the feature subset selection process and hyperparameter tuning process. Moreover, the proposed model can be applied for an accurate and automated medical data classification process in the future. 

## 4. Conclusions

In this study, a new technique has been developed for medical data classification to be applied in the EDs of the KSA hospitals and the model is abbreviated as the MLMDMC-ED technique. The most important aim of the proposed technique is to monitor and track the patient’s visits to the EDs and record the treatment provided to them at the hospital based on CTAS and LOS norms that suit the patient’s clinical requirements. The presented model exploited the NSGA II technique to choose the features. For the purpose of classification, the GWO is employed with the GCN model in this work. The proposed technique was experimentally validated using two different healthcare datasets under different measures. The experimental outcomes confirmed the superior performance of the MLMDMC-ED approach over other methods with a maximum accuracy of 91.87%. The application of the feature subset selection process and parameter tuning process helped in improving the performance of the proposed model. In the future, the experimental results of the proposed technique can be validated using large-scale real-time datasets. Besides, the proposed model can also be extended to the design of medical data classification models in Internet of Things (IoT) and cloud environments. 

## Figures and Tables

**Table 1:** Dataset details.

Class	No. of Instances	
	Cleveland Dataset	Statlog Dataset
Absence	160	150
Presence	137	120
Total No. of Instances	297	270

**Table 2:** Analytical results of the MLMDMC-ED algorithm under distinct measures on the Cleveland dataset.

Cleveland Dataset					
Class	Accuracy_bal_	Precision	Recall	F-Score	MCC
Training Phase (80%)					
Absence	100.00	81.29	100.00	89.68	77.49
Presence	73.87	100.00	73.87	84.97	77.49
Average	86.94	90.65	86.94	87.33	77.49
Testing Phase (20%)					
Absence	97.06	86.84	97.06	91.67	80.03
Presence	80.77	95.45	80.77	87.50	80.03
Average	88.91	91.15	88.91	89.58	80.03
Training Phase (70%)					
Absence	95.45	88.24	95.45	91.70	81.78
Presence	85.57	94.32	85.57	89.73	81.78
Average	90.51	91.28	90.51	90.72	81.78
Testing Phase (30%)					
Absence	100.00	84.75	100.00	91.74	81.04
Presence	77.50	100.00	77.50	87.32	81.04
Average	88.75	92.37	88.75	89.53	81.04

**Table 3:** Analytical results of the MLMDMC-ED algorithm under distinct measures on the Statlog dataset.

Statlog Dataset					
Class	Accuracybal	Precision	Recall	F-Score	MCC
Training Phase (80%)					
Absence	89.92	82.31	89.92	85.94	67.27
Presence	76.29	86.05	76.29	80.87	67.27
Average	83.10	84.18	83.10	83.41	67.27
Testing Phase (20%)					
Absence	96.77	90.91	96.77	93.75	84.93
Presence	86.96	95.24	86.96	90.91	84.93
Average	91.87	93.07	91.87	92.33	84.93
Training Phase (70%)					
Absence	85.85	96.81	85.85	91.00	81.62
Presence	96.39	84.21	96.39	89.89	81.62
Average	91.12	90.51	91.12	90.44	81.62
Testing Phase (30%)					
Absence	81.82	100.00	81.82	90.00	82.02
Presence	100.00	82.22	100.00	90.24	82.02
Average	90.91	91.11	90.91	90.12	82.02

**Table 4:** Accuracy analysis outcomes of the MLMDMC-ED algorithm and other recent algorithms [29].

Methods	Accuracy (%)
MLMDMC-ED	91.87
VNB-LR Model	87.71
Fuzzy-NN Model	80.36
DT Model	81.04
ELM Model	86.87
SVM Model	86.42
NB Model	69.31
CART Model	83.23
GA-NN Model	80.90
DT-GR Model	84.49

## Data Availability

Data sharing is not applicable to this article as no datasets were generated during the current study.
